# Adequacy of prenatal care in the state of Rio de Janeiro according to the type of childbirth funding

**DOI:** 10.11606/s1518-8787.2025059006486

**Published:** 2025-10-20

**Authors:** Rosa Maria Soares Madeira Domingues, Marcos Augusto Bastos Dias, Ana Paula Esteves-Pereira, Barbara Vasques da Silva Ayres, Alessandra do Nascimento Bernardo, Maria do Carmo Leal

**Affiliations:** I Fundação Oswaldo Cruz. Instituto Nacional de Infectologia Evandro Chagas. Laboratório de Pesquisa Clínica em HIV, Aids, Infecções Sexualmente Transmissíveis e Hepatites. Rio de Janeiro, RJ, Brasil; II Fundação Oswaldo Cruz. Instituto Nacional de Saúde da Mulher, da Criança e do Adolescente Fernandes Figueira. Departamento de Obstetrícia. Rio de Janeiro, RJ, Brasil; III Fundação Oswaldo Cruz. Escola Nacional de Saúde Pública Sérgio Arouca. Departamento de Epidemiologia e Métodos Quantitativos em Saúde. Rio de Janeiro, RJ, Brasil

**Keywords:** Prenatal Care, Health Evaluation, Cross-Sectional Studies, Maternal and Child Health, Healthcare Financing, Brazil

## Abstract

**OBJECTIVE:**

To evaluate the adequacy of prenatal care (PNC) in the state of Rio de Janeiro (SRJ) according to the type of childbirth funding.

**METHODS:**

A cross-sectional, hospital-based study conducted from 2021 to 2023 through interviews with postpartum women and collection and analysis of data from prenatal cards and medical records in public and private hospitals. Overall adequacy and adequacy of various PN components were estimated based on care guidelines from the World Health Organization and the Brazilian Ministry of Health, using 95% as the standard for adequacy.

**RESULTS:**

PN coverage was 98.5%, with 98.6% of women having received a prenatal card. Among the 1,325 women with an available card, 79,3% began PNC by the 12^th^ gestational week; 75.5% had the adequate number of consultations for gestational age at delivery; 64.7% had documentation of all first routine PN tests, and 18.9% of the second; 31.6% received adequate immunization for tetanus and hepatitis B; 29.4% received iron and folic acid supplementation; and 17.6% received counseling on delivery types, reference maternity hospital, and were asked about alcohol use and smoking. A decrease in PN adequacy was observed when all components were considered, with less than 1% of women achieving overall adequacy. Women with publicly financed births had greater social vulnerability and lower PN coverage and adequacy in terms of timing, number of consultations, tests, and counseling.

**CONCLUSION:**

PNC was found to be inadequate in SRJ, with lower adequacy among women with public financing, who represent a group with higher social vulnerability, increasing the likelihood of adverse outcomes in this population. It is essential to develop and implement strategies to improve PN adequacy and to ensure the best care for those who need it most.

## INTRODUCTION

Prenatal care (PNC) is a critical component of maternal health, with evidence supporting the effectiveness of interventions during pregnancy in reducing adverse maternal and perinatal outcomes^1,2^. In 2016, the World Health Organization (WHO) issued antenatal care guidelines aimed at promoting a positive pregnancy experience, incorporating recommendations based on the best available scientific evidence and the contextual realities of local health services^3^.

In Brazil, prenatal care coverage is nearly universal when considering the occurrence of at least one consultation during pregnancy. However, analyses of specific components, such as the timing of initiation, the number of consultations, and the tests and procedures performed, reveal shortcomings in the quality of care^4–7^.

The state of Rio de Janeiro (SRJ), the third most populous in the country, exhibits maternal mortality, congenital syphilis incidence, and fetal and perinatal mortality rates that exceed the national average^8^. These outcomes are inconsistent with the state's level of economic development^9^ and its high rates of prenatal and childbirth care coverage, suggesting deficiencies in the quality of care.

Previous studies that assessed only the timing of initiation and/or the number of prenatal consultations recorded in the Live Birth Information System (*Sistema de Informações de Nascido Vivo –* SINASC) reported adequacy rates ranging from 52 to 80% in RJ during the period 2000–2010^10–12^. However, a study conducted in the city of Rio de Janeiro (CRJ) between 2007 and 2008, which evaluated additional components of prenatal care, such as clinical procedures, supplementation, and educational activities, estimated an overall adequacy rate of only 33.3%^13^.

No studies were identified that assessed PNC in SRJ in a comprehensive manner, including potential differences in care based on the type of public or private financing. Such analyses could inform the development of strategies to improve care for pregnant women. This study aimed to evaluate the adequacy of prenatal care in the state of Rio de Janeiro according to the type of childbirth financing.

## METHODS

This study is part of the research project "Nascer no Brasil II: pesquisa nacional sobre aborto, parto e nascimento (NBII)".

### Brief Description of NBII

This is a cross-sectional, hospital-based study conducted between 2021 and 2025. A probabilistic sample was selected using a two-stage sampling design:

- hospitals;
- women and their newborns.

Hospitals were stratified by macroregion, location (metropolitan or interior), type (public, mixed, or private), and size (100–499, ≥ 500 live births [LB]/year). A total of 30 postpartum women were interviewed in hospitals with 100–499 LB/year, and 50 in those with ≥ 500 LB/year. The number of women who experienced abortion corresponded to the number of hospitalizations recorded until the target sample size of postpartum women was reached in each hospital.

Eligible participants included women who had recently given birth to a newborn of any weight or gestational age (GA), women who experienced stillbirths with GA ≥ 22 weeks or birth weight ≥ 500 g and women hospitalized with a diagnosis of miscarriage. Women were excluded if they gave birth outside a hospital; had a multiple pregnancy involving triplets or more; had communication difficulties (including foreigners, Indigenous women who did not understand Portuguese, individuals who were deaf/mute, or those with severe mental illness); were hospitalized with a diagnosis of miscarriage but discharged while still pregnant; or were hospitalized for delivery under court order.

For all eligible women, interviews were conducted during the immediate postpartum period. When available, photographs were taken and data were extracted from prenatal cards, along with clinical information obtained from hospital records. The NBII study protocol is published in Leal et al.^14^

### Study in the State of Rio de Janeiro

The sample of postpartum women in SRJ was calculated using the same parameters as the national study: the proportion of cesarean sections (57% in SRJ in 2019), a significance level of 5%, and 90% power to detect differences of 7%. A design effect of 1.3 was applied, resulting in a minimum required sample of 1,350 postpartum women. To achieve this target, the sample size was increased from 50 to 90 postpartum women in public and mixed hospitals with ≥ 500 LB/year. Hospital interviews were conducted between November 2021 and June 2023 across 29 hospitals in 18 municipalities. In total, 1,923 women were interviewed, including 1,762 postpartum women (1,752 LB and 10 stillbirths) and 161 post-abortion cases.

For the present analysis, women hospitalized due to abortion were excluded, as they did not experience a full-term pregnancy, as well as those without a typed prenatal care card.

To assess the adequacy of PNC, the following indicators were used, based on the care guidelines established by the WHO^3^ and the Brazilian Ministry of Health^15^:

- PNC: having received at least one prenatal consultation;
- Receiving the prenatal card during PNC;
- Early initiation: having had the first PN consultation by the 12^th^ gestational week;
- Adequacy of the number of consultations: having the appropriate number of visits for GA at delivery following the schedule below^3^:
  - GA ≥ 20 and < 26 = visits ≥ 2;
  - GA ≥ 26 and < 30 = visits ≥ 3;
  - GA ≥ 30 and < 34 = visits ≥ 4;
  - GA ≥ 34 and < 36 = visits ≥ 5;
  - GA ≥ 36 and < 38 = visits ≥ 6;
  - GA ≥ 38 and < 40 = visits ≥ 7;
  - GA ≥ 40 = visits ≥ 8.
- Adequacy of tests: two hemoglobin/hematocrit (Hg/Ht) measurements, two urine tests (abnormal elements and sediments or urine culture), two blood glucose tests, two syphilis serologies, and two HIV serologies, with the first test performed at the start of PNC and the second starting at 28 weeks of pregnancy, and one obstetric ultrasound (USG)^15^. The adequacy of the first and second routine for each test was analyzed separately. For the second routine, only women with GA ≥ 34 weeks at delivery were considered. The absence of recorded results was classified as non-performance of the test;
- Adequacy of counseling: having received information about the advantages and disadvantages of vaginal delivery and cesarean section, about the referral maternity hospital for delivery hospitalization, and having been asked about smoking and alcohol use during pregnancy;
- Adequacy of immunization: having been immunized against tetanus and hepatitis B. For tetanus vaccination, adequacy was defined as the record of three doses during pregnancy, a booster dose, or a dose of DTaP vaccine. For hepatitis B vaccination, adequacy was defined as a record of prior immunization or three doses administered during pregnancy^16^. Cards with no record of previous vaccination or doses received during the current pregnancy were classified as "no information;"
- Adequacy of supplementation: having received iron sulfate and folic acid supplementation during pregnancy;
- Overall adequacy: meeting all the previously listed criteria except for receiving the prenatal care card, since only women with a digitized card were included in this analysis.

Data on maternal characteristics, receipt of the prenatal card, timing of PNC initiation, number of consultations, and guidance received were obtained through interviews conducted with the women during the immediate postpartum period. Information on examinations, vaccinations, and supplementation was extracted from the PNC cards.

### Data Analysis

All analyses were conducted for the total sample and stratified by type of childbirth financing: public (births in public hospitals or in private hospitals funded by the Unified Health System (*Sistema Único de Saúde* — SUS)) and private (births in private hospitals paid for through health insurance or out-of-pocket expenditure).

Initially, the demographic and social characteristics of the women included in the analysis were described, followed by the estimation of PNC adequacy indicators with their respective 95% confidence intervals.

For all adequacy indicators, the reference standard was set at 95%, as recommended by the WHO for process indicators related to the prevention of vertical transmission of syphilis and HIV^17^; this threshold was also adopted for the other indicators evaluated in this study.

All analyses were conducted using IBM SPSS Statistics for Windows, Version 19.0 (IBM Corp., Armonk, NY, USA), incorporating the design effect, weighting, and sample calibration. For the SRJ sample, calibration was performed using groups defined by the combination of stratum, mode of delivery (vaginal or cesarean section), and maternal age (10–19, 20–34, ≥ 35 years), with the 2022 SINASC data serving as the reference.

The NBII study was approved by the National Research Ethics Commission, Certificate of Presentation for Ethical Appreciation (*Certificado de Apresentação para Apreciação Ética* – CAAE: 21633519.5.0000.5240) on March 11^th^, 2020, as well as by local institutions, prior to the initiation of fieldwork.

## RESULTS

**Figure 1:**
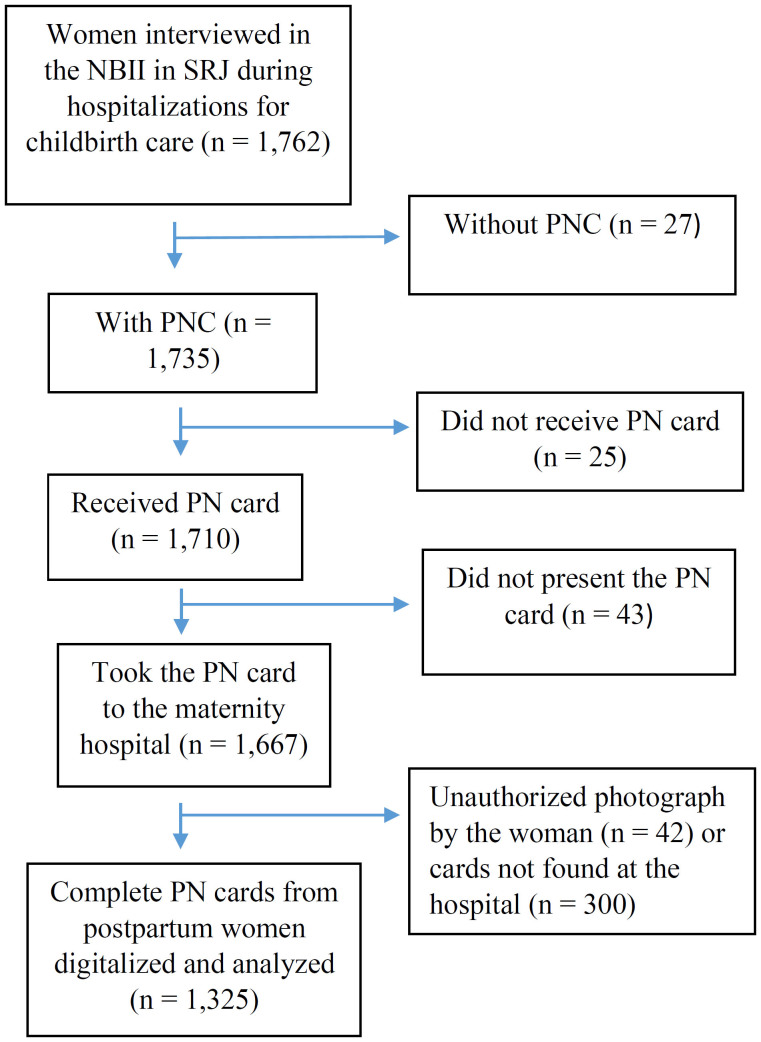
Flowchart of data collected and analyzed from postpartum women. State of Rio de Janeiro, 2021–2023.

Among the 1,762 postpartum women, PNC coverage was 98.5% (public 98%, private 99.9%, p = 0.002). The primary reason for lacking PNC was personal issues (73.5%), such as unplanned pregnancies, with 5.7% of women with public funding citing difficulties in accessing services. Of the women who received PNC, 98.6% reported receiving a prenatal card (public 99.6%, private 95.7%, p < 0.001), 97.5% brought the card to the maternity ward upon admission for delivery (public 98.5%, private 94.5%, p < 0.001), and 79.4% had the card digitized (public 85.5%, private 61.1%, p < 0.001). The main reasons for missing data were that the card was unavailable during the interview with the postpartum woman (n = 300) or that the photograph was not authorized by the woman (n = 42). After excluding ineligible women, data from 1,325 women were analyzed (Figure 1).

NBII: Nascer no Brasil II: pesquisa nacional sobre aborto, parto e nascimento.

SRJ: state of Rio de Janeiro.

PN: prenatal.

Among women with digitized cards, 81% had public funding for hospitalization during childbirth. The majority of the women were aged 20 to 34 years, identified as mixed race, had 12 to 15 years of education, lived with a partner, and were not employed. A higher proportion of postpartum women under 20 years of age, of mixed race or black, and with up to 11 years of education was observed among those with public funding. In contrast, women with private funding were more likely to be aged 35 years old or older, white, have 16 or more years of education, live with a partner, and have paid employment (Table 1).

**Table 1:** Sociodemographic characteristics of postpartum women according to the type of childbirth funding. State of Rio de Janeiro, 2021–2023.

Characteristic^a^		Total (N = 1,325) % (95%CI)	Public funding^b^ (N = 1,073) % (95%CI)	Private funding^b^ (N = 252) % (95%CI)
**Maternal age (years)**				
	< 20	11.3 (9.0–14.2)	13.6 (11.5–16.1)	1.6 (0.7–3.7)
	20 to 34	72.5 (69.6–75.2)	73.9 (70.2–77.2)	66.7(59.7–73.1)
	35 and more	16.2 (14.0–18.7)	12.5 (10.8–14.6)	31.6 (25.0–39.2)
**Skin color**				
	White	28.1 (23.4–33.4)	23.9 (18.8–29.8)	46.2 (39.8–52.7)
	Black	21.5 (17.7–25.8)	24.2 (20.5–28.2)	9.9 (7.4–13.0)
	Brown	50.4 (45.9–54.8)	51.9 (46.3–57.5)	43.9 (37.1–51.0)
**Years of study**				
	≤ 8	16.2 (13.2–19.6)	19.7 (16.7–23.2)	0.8 (0.4–1.9)
	9 to 11	30.1 (23.4–37.8)	34.8 (28.6–41.5)	10.1 (6.1–16.3)
	12 to 15	42.2 (36.4–48.2)	40.8 (34.4–47.6)	48.1 (41.6–54.6)
	16 or more	11.6 (8.2–16.1)	4.7 (3.3–6.5)	41.0 (32.1–50.5)
**Lives with a partner**		79.5 (74.8–83.5)	76.1 (72.1–79.7)	94.1 (86.8–97.5)
**Paid work**		44.6 (41.6–47.6)	39.3 (36.4–42.4)	67.1 (58.0–75.0)

95%CI: 95% confidence interval.

a Data refers only to women whose PN cards were digitized.

b Public funding: women who gave birth in a public hospital or in a private hospital with hospitalization covered by the Brazilian Unified Health System (*Sistema Único de Saúde* – SUS); private funding: women who gave birth in a private hospital with payment through health insurance plans or out-of-pocket expenses.

PNC initiation by the 12^th^ gestational week was observed in 79,3% of women. Additionally, 63,3% attended eight or more prenatal consultations, and 75.5% had the appropriate number of consultations based on their GA at delivery. The primary reasons for late initiation of PNC were personal issues (78.5%) and organizational barriers related to the healthcare service (9.3%), with no differences observed based on the type of financing. Women with private financing had earlier initiation of PNC, attended a greater number of consultations, and demonstrated better adequacy of consultations based on GA at delivery (Table 2).

**Table 2:** Indicators of prenatal care utilization, according to the type of delivery funding. State of Rio de Janeiro, 2021–2023.

Prenatal care (PNC)^a^		Total (N = 1,325) % (95%CI)	Public funding (N = 1,073)^b^ % (95%CI)	Private funding (N = 252)^b^ % (95%CI)
**Start of PNC (weeks)**				
	Up to 12	79.3 (75.9–82.3)	75.4 (71.2–79.1)	95.7 (92.6–97.5)
	13 to 27	19.3 (16.5–22.4)	22.8 (19.8–26.2)	4.3 (2.5–7.4)
	≥ 28	1.4 (0.7–3.0)	1.8 (0.8–4.0)	–––
**Reason for starting > 12 weeks** c				
	Personal issues	78.5 (73.3–83.0)	78.8 (73.3–83.3)	73.3 (46.9–89.5)
	Access barriers	4.0 (1.8–8.6)	3.9 (1.7–8.8)	5.3 (0.9–25.9)
	Organizational barriers	9.3 (6.7–13.0)	9.1 (6.3–12.8)	15.8 (3.5–48.8)
	Other reasons	8.1 (5.4–12.1)	8.2 (5.4–12.3)	5.6 (1.1–23.7)
**Number of consultations**				
	1 to 3	4.2 (3.1–5.6)	5.2 (3.9–6.8)	0.1 (0.0–0.8)
	4 to 7	32.5 (28.8–36.4)	36.4 (31.5–41.6)	16.7 (11.0–24.5)
	8 or more	63.3 (59.0–67.5)	58.4 (52.7–63.9)	83.2 (75.4–88.9)
**Adequate number of consultations according to GA at delivery** d		75.5 (72.4–78.3)	71.4 (68.0–74.6)	92.8 (86.3–96.3)

95%CI: 95% confidence interval.

a Data refers only to women whose PN cards were digitized.

b Public funding: women who gave birth in a public hospital or in a private hospital with hospitalization covered by the Brazilian Unified Health System (*Sistema Único de Saúde* – SUS); private funding: women who gave birth in a private hospital with payment through health insurance plans or out-of-pocket expenses. GA: Gestational age.

c In women with initiation > 12 weeks (public n = 251; private n = 11; total n = 262).

d Considering the schedule recommended by the World Health Organization (2016).

Approximately 80% of the prenatal care cards contained records of laboratory tests from the first routine PN visit, and 95.7% documented at least one USG during pregnancy. However, the recording of results from the second routine of tests was low, observed in approximately half of the cards. A higher proportion of women with public funding had testing for syphilis and HIV during the first routine, while women with private funding had a higher proportion of two tests for Hg/Ht, HIV serology, blood glucose, and urine analysis. Complete documentation of the first routine was observed in 64.7% of the cards, 18.9% for the second routine, with a higher proportion of complete second routine records among women with private funding (Table 3).

**Table 3:** Exams, vaccinations, supplements, and guidance during prenatal care, according to the type of delivery financing. State of Rio de Janeiro, 2021–2023.

Procedure^a^		Total (N = 1,325) % (95%CI)	Public funding^b^ (N = 1,273) % (95%CI)	Private funding^b^ (N = 252) % (95%CI)
**Exams**				
	One Hg/Ht	77.7 (72.6–82.1)	78.1 (72.2–83.0)	76.2 (69.2–82.0)
	One syphilis	84.6 (79.0–88.9)	86.7 (80.8–91.0)	75.6 (68.7–81.3)
	One anti-HIV	81.2 (75.8–85.7)	82.6 (76.4–87.4)	75.6 (68.8–81.4)
	One blood sugar test	77.9 (73.3–82.0)	78.4 (73.2–82.9)	75.8 (68.7–81.6)
	One urine test	74.9 (69.9–79.4)	75.2 (69.2–80.3)	73.9 (67.3–79.7)
	One USG	95.7 (94.4–96.7)	95.6 (94.2–96.7)	96.1 (92.7–97.9)
**First routine adequacy**		64.7 (59.2–69.8)	64.8 (58.3–70.7)	64.4 (55.0–72.8)
	Two Hg/Ht^c^	51.6 (45.6–57.6)	49.0 (41.6– 56.5)	62.5 (50.2–73.3)
	Two syphilis tests^c^	50.1 (44.0–58.0)	49.7 (40.9–58.4)	56.8 (47.5–65.6)
	Two anti-HIV tests^c^	45.5 (38.8–52.3)	42.9 (34.7–51.4)	56.4 (46.9–65.5)
	Two blood sugar tests^c^	37.9 (33.8–42.2)	34.8 (29.5–40.6)	50.8 (43.6–57.9)
	Two urine tests^c^	43.2 (36.9–49.8)	40.6 (32.8–49.1)	54.0 (43.3–64.4)
**Second routine adequacy** c		18.9 (15.8–22.5)	15.2 (12.6–18.2)	34.6 (25.8–44.6)
**Tetanus vaccination**				
	Adequate immunization	59.9 (52.1–67.2)	61.5 (52.5–69.8)	52.9 (46.6–59.1)
	Inadequate immunization	5.0 (2.7–9.2)	5.4 (2.5–11.0)	3.7 (2.0–6.7)
	No information	35.1 (29.7–40.9)	33.2 (27.3–39.6)	43.4 (36.4–50.6)
**Hepatitis B vaccination**				
	Adequate immunization	35.8 (28.8–43.4)	36.1 (27.9–45.2)	34.5 (26.6–43.3)
	Inadequate immunization	15.2 (11.8–19.4)	16.6 (12.0–22.4)	9.5 (6.2–14.4)
	No information	49.0 (42.8–55.2)	47.3 (40.3–54.5)	56.0 (47.4–64.3)
**Vaccination adequacy**		31.6 (23.6–40.9)	31.8 (22.4–43.0)	30.6 (23.4–39.0)
	Iron sulfate supplementation	39.8 (31.0–49.3)	43.2 (31.5–55.8)	25.2 (14.9–39.3)
	Folic acid supplementation	33.7 (26.7–41.5)	36.4 (27.5–46.4)	22.3 (13.0–35.6)
**Supplementation adequacy**		29.4 (22.0–38.1)	31.7 (22.3–42.9)	19.9 (11.2–32.8)
**Guidance**				
	Risks and benefits of vaginal delivery	50.6 (44.3–56.9)	45.1 (39.2–51.2)	74.0 (58.1–85.4)
	Risks and benefits of cesarean section	48.6 (42.2–55.0)	42.3 (36.7–48.0)	75.7 (62.5–85.3)
	Reference maternity hospital	66.2 (55.1–75.7)	63.4 (50.3–74.8)	72.3 (53.4–85.6)
	Tobacco use screening	68.7 (60.6–75.8)	67.4 (58.0–75.5)	74.3 (62.1–83.6)
	Alcohol use screening	72.6 (64.8–79.3)	71.0 (61.9–78.7)	79.6 (68.0–87.7)
**Guidance adequacy**		17.6 (10.8–27.2)	11.9 (7.2–18.8)	41.8 (23.4–62.9)
**Overall adequacy** d		0.6 (0.3–1.5)	0.5 (0.2–1.5)	1.1 (0.3–4.2)

95%CI: 95% confidence interval. USG: ultrasound.

a Data refers only to women whose PN cards were digitized.

b Public funding: women who gave birth in a public hospital or in a private hospital with hospitalization covered by the Brazilian Unified Health System (*Sistema Único de Saúde* – SUS); private funding: women who gave birth in a private hospital with payment through health insurance plans or out-of-pocket expenses.

c In women with a gestational age ≥ 34 weeks (public n = 1,047; private n = 251; total n = 1,298).

d Women with prenatal care initiation up to 12 weeks gestational age; the appropriate number of consultations for the gestational age at delivery; all routine exams (second routine only for women with gestational age ≥ 34 weeks); vaccination against tetanus and hepatitis B; supplementation with iron sulfate and folic acid; and counseling on smoking, alcohol, type of delivery, and reference maternity hospital for delivery admission.

The proportion of pregnant women with no vaccination information was 35.1% for tetanus and 49.0% for hepatitis B. Adequate immunization for tetanus was recorded in 59.9% of the cards, while 35.8% documented adequate immunization for hepatitis B, with no significant differences observed based on the type of financing (Table 3). Iron sulfate supplementation was prescribed in 39.8% of the cards, and folic acid in 33.7%, with lower prescription rates noted among women with private financing (Table 3). Half of the women reported receiving guidance on the risks and benefits of vaginal delivery *versus* cesarean section, and 66.2% were advised on the reference maternity hospital for childbirth care. Approximately 70% of the women indicated they were asked about smoking and alcohol consumption during pregnancy. Women with private financing received more guidance on the risks and benefits of delivery options, but there were no significant differences regarding other types of guidance provided (Table 3).

**Figure 2:**
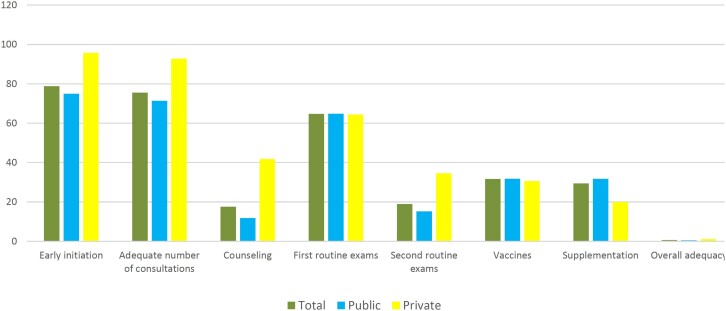
Adequacy of prenatal care according to the type of childbirth funding. State of Rio de Janeiro, 2021–2023.

The adequacy of PNC varied from 79,3% (timing of initiation) to 17.6% (guidance). When all components were assessed collectively, the overall adequacy was found to be only 0.6% among the postpartum women, with no significant differences based on the type of financing (Figure 2 and Table 3).

## DISCUSSION

The results of this study reveal a low level of PNC adequacy in the SRJ, with only the indicators "at least one PN visit," "receipt of a PN card," and "one USG examination during pregnancy" exceeding 95%, the reference standard. When assessed using a set of criteria recommended by both the WHO and the Brazilian Ministry of Health, adequacy was observed in less than 1% of pregnant women. The findings also highlight the social inequalities present in the state, with a higher proportion of pregnant adolescents, black and brown women, those with fewer years of education, and those without a partner or paid employment among women with public funding. These inequalities are further reflected in disparities in access to and utilization of prenatal services, with lower PNC coverage, later initiation, inadequate consultation frequency, and reduced access to guidance and exams. Social and demographic inequalities in PNC adequacy have also been observed in other Brazilian studies, with lower adequacy reported among black women^12,18,19^, adolescents^7,12,19^, and those with lower education^7,12^ and income^4,7^.

No previous studies evaluating the adequacy of PNC in the SRJ were identified. While not an ideal comparison, data from a nationwide study conducted in 2011/2012^20^, which estimated results for the Southeast region, where SRJ is located, reveal an increase in the proportion of postpartum women who brought their pregnancy card to the maternity hospital — from 78.2% in the Southeast region to 97,5% in this study. A lower rate of card receipt and presentation at the maternity hospital was observed among women with private financing, likely due to the predominant care model in the private sector, where PNC and delivery are generally managed by the same healthcare professional^21^.

There was also an increase in the proportion of pregnant women who initiated PNC by the 12^th^ week of gestation, compared to data from 2011/2012. This figure rose from 58.2% in the Southeast region to 79.3% in this study^5^. When considering the type of financing, a comparison of national data with data from this study shows an increase in early prenatal care initiation from 51.6 to 75.4% in the public sector, and from 60.3 to 95.7% in the private sector^5^. Data from SINASC indicate an increase in early PNC initiation in SRJ from 2012 to 2022, rising from 65.6 to 78.1%, with the national average being 80.8%^8^.

The comparison of the adequacy of the number of consultations with other studies is limited by the criterion used in this study, which required at least eight consultations for a normal-risk pregnant woman, whereas previous studies generally used a threshold of six visits. In 2011/2012, 73.1% of pregnant women had at least six consultations^20^, while in the third evaluation cycle of the Access and Quality Improvement Program (*Programa de Melhoria do Acesso e da Qualidade* – PMAQ), conducted in 2017/2018, this figure was 77.8%^6^. In this study, 63.3% of women reported having eight or more visits, representing a lower adequacy, but with a more demanding criterion. Notably, 75.5% of women had the appropriate number of consultations based on GA at delivery. Data from SINASC show an increase in the proportion of women in SRJ with more than seven consultations between 2012 and 2022, rising from 54.9 to 64.3%, with the national average being 63.7%^8^.

With the exception of USG, none of the routine tests reached the recommended adequacy, with negative implications for the timely diagnosis and treatment of conditions that impact maternal and perinatal outcomes, such as syphilis, HIV infection, urinary tract infections, diabetes, and anemia. Even lower adequacy was observed for the second set of routine tests, assessed among women who delivered at 34 or more weeks of gestation. The higher adequacy observed among women with private financing, also reported in a previous study^5^, likely reflects greater access to laboratory services in the private sector. The adequacy of tests, excluding USG, was similar to that reported in a national study conducted in 2011/2012^20^ and lower than that observed in the third evaluation cycle of the PMAQ^6^. It is important to note that the latter relied on interviews with mothers of children under two years of age, making the findings more susceptible to recall bias. Compared to a study conducted with pregnant women receiving PN care in public units in CRJ^13^, an increase was observed in the proportion of women with public funding who underwent the first urine test and HIV serology, as well as the second urine test and syphilis serology.

Vaccination during pregnancy is a key strategy to prevent neonatal tetanus and the vertical transmission of hepatitis B. Among women with public funding, tetanus vaccination coverage was lower than that reported in the third PMAQ evaluation cycle^6^ and in a study involving pregnant women receiving care in public units in CRJ^13^, but similar to that observed in a study conducted in Espírito Santo^4^. It is important to note the high proportion of prenatal cards with no recorded information. No previous studies were identified that specifically assessed hepatitis B vaccination in pregnant women, with adequacy observed in only one-third of postpartum women evaluated in the present study. In a study conducted in João Pessoa/Paraíba, adequate vaccination coverage for tetanus, hepatitis B, and influenza was reported in 71% of pregnant women^7^.

Iron and folic acid supplementation during pregnancy has demonstrated beneficial effects in low- and middle-income populations, including a reduction in the proportion of low birth weight infants and a probable decrease in the incidence of preterm births and small-for-gestational-age newborns^22^. In this study, the adequacy of iron sulfate supplementation was considerably lower than that reported in the PMAQ^6^ and in a study conducted in public healthcare units in CRJ^13^. It is important to highlight that, in both of these previous studies, data were collected through interviews with pregnant women, whereas in the present study, information was extracted from the prenatal cards. No national studies evaluating folic acid supplementation during pregnancy were identified.

PNC represents a key opportunity for implementing educational interventions. In this study, four types of guidance were selected based on their relevance: screening for smoking and alcohol use during pregnancy, modifiable risk factors for adverse perinatal outcomes^23,24^ that can be addressed through education and support services^25^; information on the advantages and disadvantages of different delivery methods, with the aim of promoting vaginal birth in a country with one of the highest cesarean rates globally; and referral of pregnant women to a designated maternity hospital for childbirth care, as mandated by national legislation since 2007^26^. These counseling activities depend solely on the actions of healthcare professionals and may be carried out in individual or group settings. In this study, approximately two-thirds of women were screened for smoking and alcohol use and received guidance on their reference maternity hospital, while only half received counseling on childbirth. Previous studies evaluating referral to maternity hospitals^13,20^ and guidance related to childbirth (such as signs of labor or labor-facilitating activities)^7,13,20^ reported similar results. No studies in Brazil were identified that assessed screening for smoking and alcohol use during pregnancy, and the proportion of professionals reporting routine implementation of such screenings varies across countries^27–29^.

The overall adequacy of PNC was found to be less than 1%, with a marked reduction particularly after the inclusion of examinations and counseling components. Similar findings have been reported in studies that incorporated additional elements beyond the timing of initiation and number of consultations^4,5,7,13,30^, with adequacy ranging from 0.4^4^ to 38.5%^13^, although comparisons are limited due to differences in the criteria adopted. Notably, no significant differences were observed between women with public or private funding, underscoring the persistent challenges in delivering comprehensive and integrated care across both sectors.

This study has some limitations. Only women who presented a digitalized PN card were included in the analysis, resulting in the exclusion of 20% of those who presented the card at the maternity hospital. This loss was selective, being greater among women with private financing, who exhibited higher adequacy in some of the components analyzed. Therefore, the adequacy of PNC among all women in SRJ may be underestimated. Additionally, women who gave birth at home, in public spaces, or in hospitals with fewer than 100 deliveries per year were not included, and the findings do not reflect the PNC received by these groups. Data on examinations, vaccinations, and supplementation were obtained from the PN card, and although these procedures and prescriptions may have been carried out, they may not have been recorded. Nevertheless, the PN card serves as the main link between outpatient and hospital care, and the documentation of information is itself an indicator of PNC adequacy. Finally, the classification of women into public or private groups was based on childbirth care financing, which may not coincide with the type of financing for PNC, particularly among those with health plan coverage limited to outpatient services, potentially attenuating differences between the groups.

PNC in SRJ was found to be inadequate when all analyzed components were considered. The lower adequacy of several PNC elements among women with public funding, who represent a socially vulnerable population, increases the likelihood of adverse outcomes in this group. It is essential to implement targeted strategies that ensure the highest quality of care is delivered to those who need it most.

## Data Availability

The datasets generated and/or analyzed during the present study are not publicly available due to [ethical/legal/privacy] restrictions, but are available from the corresponding author upon request.
