# Supplementary material for: Adequacy of prenatal care in the state of Rio de Janeiro according to the type of childbirth funding
Source: Rev Saude Publica. 2025 Oct 20;59(Suppl 1):e5s. doi: 10.11606/s1518-8787.2025059006486 (PMC12539637; doi:10.11606/s1518-8787.2025059006486)
Supplement: Translation

# Translation: Adequação do pré-natal no estado do Rio de Janeiro segundo o tipo de financiamento do parto

**Authors:** Rosa Maria Soares Madeira Domingues, Marcos Augusto Bastos Dias, Ana Paula Esteves-Pereira, Barbara Vasques da Silva Ayres, Alessandra do Nascimento Bernardo, Maria do Carmo Leal

**Affiliations:** I Fundação Oswaldo Cruz. Instituto Nacional de Infectologia Evandro Chagas. Laboratório de Pesquisa Clínica em HIV, Aids, Infecções Sexualmente Transmissíveis e Hepatites. Rio de Janeiro, RJ, Brasil; II Fundação Oswaldo Cruz. Instituto Nacional de Saúde da Mulher, da Criança e do Adolescente Fernandes Figueira. Departamento de Obstetrícia. Rio de Janeiro, RJ, Brasil; III Fundação Oswaldo Cruz. Escola Nacional de Saúde Pública Sérgio Arouca. Departamento de Epidemiologia e Métodos Quantitativos em Saúde. Rio de Janeiro, RJ, Brasil

**Keywords:** Cuidado Pré-natal, Avaliação em Saúde, Estudos Transversais, Saúde Materno-Infantil, Financiamento da Assistência à Saúde, Brasil

## Abstract

**OBJETIVO:**

Avaliar a adequação da assistência pré-natal (PN) no estado do Rio de Janeiro (ERJ) segundo o tipo de financiamento do parto.

**MÉTODOS:**

Estudo transversal, de base hospitalar, realizado no período 2021–2023, por meio de entrevista com puérperas, coleta e análise de dados de cartão da gestante e prontuário médico em hospitais públicos e privados. Foi estimada a adequação global e dos diversos componentes da assistência PN, utilizando como critério as diretrizes assistenciais da Organização Mundial da Saúde e Ministério da Saúde brasileiro, com o parâmetro de 95% como padrão de adequação.

**RESULTADOS:**

Cobertura PN de 98,5%, com 98,6% das mulheres tendo recebido cartão da gestante. Das 1.325 mulheres com cartão disponível, 79,3% tiveram início da assistência pré-natal até a 12ª semana gestacional; 75,5% tiveram o número adequado de consultas para a idade gestacional no parto; 64,7% tiveram o registro de todos os exames da primeira rotina de PN e 18,9% da segunda; 31,6% receberam imunização adequada para tétano e hepatite B; 29,4% receberam suplementação de sulfato ferroso e ácido fólico; e 17,6% receberam orientações sobre tipos de parto, maternidade de referência e foram questionadas sobre o uso de álcool e tabagismo. Verificou-se redução da adequação do PN ao incorporar todos os componentes, com menos de 1% das mulheres apresentando adequação global do PN. Mulheres com financiamento público apresentaram maior vulnerabilidade social e menor cobertura de PN e de adequação de época de início, número de consultas, exames e orientações.

**CONCLUSÃO:**

A assistência pré-natal mostrou-se inadequada no ERJ, com menor adequação em mulheres com financiamento público, que constituem um grupo com maior vulnerabilidade social, aumentando a probabilidade de desfechos negativos nessa população. É essencial a elaboração e implantação de estratégias destinadas a aumentar a adequação do PN e oferecer o melhor cuidado justamente àquelas que mais necessitam dessas ações.

## INTRODUÇÃO

A assistência pré-natal (PN) é um importante componente da atenção à saúde materna, havendo evidências do benefício de intervenções realizadas durante a gestação para a redução de desfechos maternos e perinatais negativos^1,2^. Em 2016, a Organização Mundial da Saúde (OMS) publicou as diretrizes assistenciais para uma assistência pré-natal voltada para uma experiência positiva da gravidez, com recomendações baseadas nas melhores evidências científicas e no contexto local dos serviços^3^.

No Brasil, a cobertura do PN é praticamente universal, quando considerada a realização de pelo menos uma consulta durante a gestação. Entretanto, a análise de seus componentes, incluindo a época de início, o número de consultas e os exames e procedimentos realizados indicam falhas nessa assistência^4–7^.

O estado do Rio de Janeiro (ERJ) é o terceiro mais populoso do país e apresenta elevada razão de mortalidade materna, taxa de incidência de sífilis congênita e taxas de mortalidade fetal e perinatal superiores à média nacional^8^. Esses resultados não são compatíveis com o desenvolvimento econômico do estado^9^ nem com a elevada cobertura pré-natal e ao parto, sugerindo falhas na qualidade da assistência.

Estudos anteriores, que avaliaram apenas a época de início e/ou o número de consultas de PN registrados no Sistema de Informações de Nascido Vivo (SINASC), encontraram proporção de adequação que variou de 52 a 80% no ERJ no período 2000–2020^10–12^. Entretanto, um estudo realizado no município do Rio de Janeiro (MRJ) em 2007–2008, que avaliou outros componentes da assistência pré-natal, como procedimentos clínicos, suplementações e atividades educativas, estimou adequação global de apenas 33,3%^13^.

Não foram identificados estudos que tenham avaliado a assistência PN no ERJ de forma mais abrangente, incluindo possíveis diferenças entre a assistência segundo o tipo de financiamento público ou privado, que possam orientar a formulação de estratégias de melhoria do cuidado à gestante. Este estudo tem por objetivo avaliar a adequação da assistência pré-natal no ERJ segundo o tipo de financiamento do parto.

## MÉTODOS

Este estudo é parte da pesquisa "Nascer no Brasil II: pesquisa nacional sobre aborto, parto e nascimento (NBII)".

### Breve Descrição da NBII

Trata-se de estudo transversal, de base hospitalar, realizado no período 2021–2025. Foi selecionada uma amostra probabilística em dois estágios:

- hospitais;
- mulheres e seus recém-nascidos.

Os hospitais foram estratificados por macrorregião, localização (região metropolitana ou interior), tipo (público, misto, privado) e tamanho do hospital (100–499, ≥ 500 nascidos vivos [NV]/ano). Foram entrevistadas 30 e 50 puérperas nos hospitais com 100–499 e ≥ 500 NV/ano, respectivamente. O número de mulheres com abortamento correspondeu ao número de internações até atingir a amostra prevista de puérperas em cada hospital.

Foram elegíveis puérperas de NV com qualquer peso ou idade gestacional (IG), puérperas de natimortos com IG ≥ 22 semanas ou peso ≥ 500 g e mulheres internadas com diagnóstico de abortamento. Não foram elegíveis mulheres com parto fora da instituição hospitalar; com parto trigemelar ou mais; com dificuldade de comunicação (estrangeiras, indígenas que não entendiam português, surdas/mudas, ou com doença mental severa); internadas com diagnóstico de abortamento, mas que receberam alta ainda gestantes; e internadas para a realização de parto por determinação judicial.

Para todas as mulheres incluídas, foram realizadas entrevistas no puerpério imediato, e foram fotografados e extraídos dados dos cartões de pré-natal, quando disponíveis, e dados clínicos do prontuário hospitalar. O protocolo do estudo NBII encontra-se publicado em Leal et al.^14^

### Estudo no Estado do Rio de Janeiro

A amostra de puérperas do ERJ foi calculada utilizando-se os mesmos parâmetros do estudo nacional: proporção de cesarianas (57% no ERJ em 2019), nível de significância de 5% e potência de 90% para detectar diferenças de 7%. Utilizou-se um efeito de desenho de 1,3, resultando no planejamento de um tamanho amostral mínimo de 1.350 puérperas. Para alcançar esse quantitativo, houve ampliação da amostra de 50 para 90 puérperas nos hospitais públicos e mistos com ≥ 500 NV/ano. As entrevistas hospitalares ocorreram entre novembro de 2021 e junho de 2023 em 29 hospitais de 18 municípios. No total, foram entrevistadas 1.923 mulheres, sendo 1.762 puérperas (1.752 NV e dez natimortos) e 161 pós-aborto.

Para a presente análise, foram excluídas as mulheres internadas por abortamento, já que não apresentaram gestação com duração completa, e as que não tiveram cartão da gestante digitado.

Para a análise da adequação do PN, foram utilizados os indicadores descritos abaixo, utilizando como referência as diretrizes assistenciais da OMS^3^ e do Ministério da Saúde brasileiro^15^:

- Assistência PN: ter recebido ao menos uma consulta de pré-natal;
- Recebimento do cartão da gestante durante a assistência PN;
- Início precoce: ter tido a primeira consulta PN até a 12ª semana gestacional;
- Adequação do número de consultas: ter o número adequado de consultas para a IG no parto seguindo o seguinte calendário^3^:
  - IG ≥ 20 e < 26 = consultas ≥ 2;
  - IG ≥ 26 e < 30 = consultas ≥ 3;
  - IG ≥ 30 e < 34 = consultas ≥ 4;
  - IG ≥ 34 e < 36 = consultas ≥ 5;
  - IG ≥ 36 e < 38 = consultas ≥ 6;
  - IG ≥ 38 e < 40 = consultas ≥ 7;
  - IG ≥ 40 = consultas ≥ 8.
- Adequação de exames: duas dosagens de hemoglobina/hematócrito (Hg/Ht), dois exames de urina (elementos anormais e sedimentos ou urinocultura), duas dosagens de glicemia, duas sorologias para sífilis e duas sorologias para HIV, sendo o primeiro exame no início do pré-natal e o segundo a partir da 28^a^ semana de gestação, e uma ultrassonografia obstétrica (USG)^15^. A adequação da primeira e segunda rotina de cada exame foi analisada separadamente. Para a segunda rotina, foram consideradas apenas as puérperas com IG ≥ 34 semanas no momento do parto. A ausência de registro do resultado foi classificada como não realização do exame;
- Adequação de orientação: ter recebido orientação sobre vantagens e desvantagens do parto vaginal e da cesariana, sobre a maternidade de referência para internação para o parto, e ter sido questionada sobre tabagismo e uso de álcool na gestação;
- Adequação de vacinação: ter sido imunizada para tétano e hepatite B. Para a vacinação contra o tétano, foi considerado adequado o registro de três doses da vacina durante a gestação, ou dose de reforço, ou dose de dTpa. Para a vacinação contra hepatite B, foi considerado adequado o registro de imunização prévia ou de três doses durante a gestação^16^. Cartões sem registro dos antecedentes vacinais ou doses recebidas durante a gestação atual foram classificados como "sem informação";
- Adequação de suplementação: ter recebido suplementação de sulfato ferroso e ácido fólico durante a gestação;
- Adequação total: ter atendido aos critérios listados anteriormente exceto o recebimento de cartão, já que apenas mulheres com cartão digitado foram incluídas nesta análise.

Dados relativos às características maternas e ao recebimento de cartão, época de início do PN, número de consultas e orientações recebidas são provenientes da entrevista com a mulher no pós-parto imediato, enquanto os dados relativos a exames, vacinas e suplementações foram obtidos no cartão da gestante.

### Análise dos Dados

Todas as análises foram realizadas para a amostra total e categorizadas segundo o tipo de financiamento do parto: público (hospital público ou hospital privado com internação paga pelo Sistema Único de Saúde — SUS) e privado (hospital privado com pagamento da internação por plano de saúde ou desembolso direto).

Inicialmente, foram analisadas as características demográficas e sociais das mulheres incluídas na análise e, posteriormente, foram estimados os indicadores de adequação do PN com os respectivos intervalos de confiança de 95%.

Para todos os indicadores de adequação, o padrão de referência utilizado foi o valor de 95%, recomendado pela OMS para indicadores de processo para a prevenção da transmissão vertical da sífilis e do HIV^17^, também adotado neste estudo para os demais indicadores.

Todas as análises foram realizadas com o *software* IBM SPSS Statistics for Windows, Versão 19.0 (IBM Corp., Armonk, NY, USA), com inclusão do efeito de desenho, ponderação e calibração da amostra. Para a amostra do ERJ, foram utilizados para calibração grupos compostos da combinação de estrato, via de nascimento (vaginal, cesariana) e idade da mulher (10–19, 20–34, ≥ 35), utilizando o SINASC-2022 como referência.

A pesquisa NBII foi aprovada pela Comissão Nacional de Ética em Pesquisa, Certificado de Apresentação para Apreciação Ética (CAAE): 21633519.5.0000.5240, em 11 de março de 2020, e pelas instituições locais, antes do início do trabalho de campo.

## RESULTADOS

Entre as 1.762 puérperas, a cobertura de PN foi de 98,5% (público 98%, privado 99,9%, p = 0,002). O principal motivo para a não realização do PN foram questões pessoais (73,5%), tais como não ter planejado a gravidez, tendo 5,7% das mulheres com financiamento público referido dificuldades de acesso ao serviço. Das mulheres que tiveram assistência PN, 98,6% referiram ter recebido cartão da gestante (público 99,6%, privado 95,7%, p < 0,001), 97,5% levaram o cartão para a maternidade no momento da admissão para o parto (público 98,5%, privado 94,5%, p < 0,001) e 79,4% tiveram o cartão digitado (público 85,5%, privado 61,1%, p < 0,001). Os principais motivos para a não digitação foram o cartão não estar disponível no momento da entrevista com a puérpera (n = 300) ou a fotografia não ter sido autorizada pela mulher (n = 42). Após as exclusões de mulheres não elegíveis, foram analisados os dados de 1.325 mulheres (Figura 1).

**Figura 1:** Fluxograma dos dados coletados e analisados de puérperas pós-parto. Estado do Rio de Janeiro, 2021–2023.

NBII: Nascer no Brasil II: pesquisa nacional sobre aborto, parto e nascimento.

ERJ: estado do Rio de Janeiro.

PN: pré-natal.

Das mulheres com cartão digitado, 81% tiveram financiamento público da internação para o parto. A maior parte das mulheres tinha de 20 a 34 anos, era de cor/raça parda, apresentava 12 a 15 anos de estudo, vivia com companheiro e não possuía trabalho remunerado. Observou-se maior proporção de puérperas com idade inferior a 20 anos, pardas e pretas, e com até 11 anos de estudo em mulheres com financiamento público, enquanto entre aquelas com financiamento privado se observou maior proporção de mulheres com 35 anos ou mais, de cor branca, com 16 ou mais anos de estudo, que viviam com companheiro e que possuíam trabalho remunerado (Tabela 1).

**Tabela 1:** Características sociodemográficas das puérperas segundo tipo de financiamento do parto. Estado do Rio de Janeiro, 2021–2023.

Característica^a^		Total (N = 1.325) % (IC95%)	Financiamento público^b^ (N = 1.073) % (IC95%)	Financiamento privado^b^ (N = 252) % (IC95%)
**Idade materna (anos)**				
	< 20	11,3 (9,0–14,2)	13,6 (11,5–16,1)	1,6 (0,7–3,7)
	20 a 34	72,5 (69,6–75,2)	73,9 (70,2–77,2)	66,7(59,7–73,1)
	35 e mais	16,2 (14,0–18,7)	12,5 (10,8–14,6)	31,6 (25,0–39,2)
**Cor da pele**				
	Branca	28,1 (23,4–33,4)	23,9 (18,8–29,8)	46,2 (39,8–52,7)
	Preta	21,5 (17,7–25,8)	24,2 (20,5–28,2)	9,9 (7,4–13,0)
	Parda	50,4 (45,9–54,8)	51,9 (46,3–57,5)	43,9 (37,1–51,0)
**Anos de estudo**				
	≤ 8	16,2 (13,2–19,6)	19,7 (16,7–23,2)	0,8 (0,4–1,9)
	9 a 11	30,1 (23,4–37,8)	34,8 (28,6–41,5)	10,1 (6,1–16,3)
	12 a 15	42,2 (36,4–48,2)	40,8 (34,4–47,6)	48,1 (41,6–54,6)
	16 ou mais	11,6 (8,2–16,1)	4,7 (3,3–6,5)	41,0 (32,1–50,5)
**Vive com o companheiro**		79,5 (74,8–83,5)	76,1 (72,1–79,7)	94,1 (86,8–97,5)
**Trabalho remunerado**		44,6 (41,6–47,6)	39,3 (36,4–42,4)	67,1 (58,0–75,0)

IC95%: Intervalo de confiança 95%.

a Dados relativos apenas a mulheres que tiveram cartão de PN digitado.

b Financiamento público: mulheres com parto em hospital público ou privado com internação paga pelo Sistema Único de Saúde; financiamento privado: mulheres com parto em hospital privado com pagamento por planos de saúde ou desembolso direto.

O início da assistência PN até a 12ª semana gestacional foi observado em 79,3% das mulheres, 63,3% tiveram oito ou mais consultas de pré-natal e 75,5% o número adequado de consultas para a IG no parto. Os principais motivos para o início tardio da assistência PN foram questões pessoais (78,5%) e barreiras organizacionais relacionadas ao serviço (9,3%), sem diferenças segundo tipo de financiamento. Mulheres com financiamento privado apresentaram início mais precoce do PN, maior número de consultas e maior adequação do número de consultas para a IG no parto (Tabela 2).

**Tabela 2:** Indicadores de utilização da assistência pré-natal, segundo o tipo de financiamento do parto. Estado do Rio de Janeiro, 2021–2023.

Assistência pré-natal (PN)^a^		Total (N = 1.325) % (I 95%)	Financiamento público (N = 1.073)^b^ % (IC95%)	Financiamento privado N = 252)^b^ % (IC95%)
**Início PN (semanas)**				
	Até 12	79,3 (75,9–82,3)	75,4 (71,2–79,1)	95,7 (92,6–97,5)
	13 a 27	19,3 (16,5–22,4)	22,8 (19,8–26,2)	4,3 (2,5–7,4)
	≥ 28	1,4 (0,7–3,0)	1,8 (0,8–4,0)	–––
**Motivo início > 12 semanas** c				
	Questões pessoais	78,5 (73,3–83,0)	78,8 (73,3–83,3)	73,3 (46,9–89,5)
	Barreiras de acesso	4,0 (1,8–8,6)	3,9 (1,7–8,8)	5,3 (0,9–25,9)
	Barreiras organizacionais	9,3 (6,7–13,0)	9,1 (6,3–12,8)	15,8 (3,5–48,8)
	Outros motivos	8,1 (5,4–12,1)	8,2 (5,4–12,3)	5,6 (1,1–23,7)
**Nº consultas**				
	1 a 3	4,2 (3,1–5,6)	5,2 (3,9–6,8)	0,1 (0,0–0,8)
	4 a 7	32,5 (28,8–36,4)	36,4 (31,5–41,6)	16,7 (11,0–24,5)
	8 ou mais	63,3 (59,0–67,5)	58,4 (52,7–63,9)	83,2 (75,4–88,9)
**Nº adequado de consultas segundo IG no parto** d		75,5 (72,4–78,3)	71,4 (68,0–74,6)	92,8 (86,3–96,3)

IC95%: Intervalo de confiança de 95%.

a Dados relativos apenas a mulheres que tiveram cartão de PN digitado.

b Financiamento público: mulheres com parto em hospital público ou privado com internação paga pelo Sistema Único de Saúde; financiamento privado: mulheres com parto em hospital privado com pagamento por planos de saúde ou desembolso direto. IG: Idade gestacional.

c Em mulheres com início > 12 semanas (público n = 251; privado n = 11; total n = 262).

d Considerando-se o calendário preconizado pela Organização Mundial da Saúde (2016).

Aproximadamente 80% dos cartões apresentavam registro de algum dos exames laboratoriais da primeira rotina de PN e 95,7% o registro de pelo menos uma USG durante a gestação. O registro do resultado da segunda rotina de exames foi baixo, observado em aproximadamente metade dos cartões. Maior proporção de testagem da primeira rotina para sífilis e HIV foi observada em mulheres com financiamento público e maior proporção de realização de dois exames para Hg/Ht, sorologia anti-HIV, glicemia e exame de urina em mulheres com financiamento privado. A realização completa da primeira rotina foi verificada em 64,7% dos cartões e da segunda rotina em 18,9%, com maior proporção da segunda rotina em mulheres com financiamento privado (Tabela 3).

**Tabela 3:** Exames, vacinas, suplementações e orientações durante o pré-natal, segundo tipo de financiamento do parto. Estado do Rio de Janeiro, 2021–2023.

Procedimento^a^		Total (N = 1.325) % (IC95%)	Financiamento público^b^ (N = 1.273) % (IC95%)	Financiamento privado^b^ (N = 252) % (IC95%)
**Exames**				
	Um Hg/Ht	77,7 (72,6–82,1)	78,1 (72,2–83,0)	76,2 (69,2–82,0)
	Um sífilis	84,6 (79,0–88,9)	86,7 (80,8–91,0)	75,6 (68,7–81,3)
	Um anti-HIV	81,2 (75,8–85,7)	82,6 (76,4–87,4)	75,6 (68,8–81,4)
	Uma glicemia	77,9 (73,3–82,0)	78,4 (73,2–82,9)	75,8 (68,7–81,6)
	Um exame urina	74,9 (69,9–79,4)	75,2 (69,2–80,3)	73,9 (67,3–79,7)
	Uma USG	95,7 (94,4–96,7)	95,6 (94,2–96,7)	96,1 (92,7–97,9)
**Adequação primeira rotina**		64,7 (59,2–69,8)	64,8 (58,3–70,7)	64,4 (55,0–72,8)
	Dois Hg/H^c^	51,6 (45,6–57,6)	49,0 (41,6– 56,5)	62,5 (50,2–73,3)
	Dois Sífilis^c^	50,1 (44,0–58,0)	49,7 (40,9–58,4)	56,8 (47,5–65,6)
	Dois anti-HIV^c^	45,5 (38,8–52,3)	42,9 (34,7–51,4)	56,4 (46,9–65,5)
	Duas glicemias^c^	37,9 (33,8–42,2)	34,8 (29,5–40,6)	50,8 (43,6–57,9)
	Dois exames urina^c^	43,2 (36,9–49,8)	40,6 (32,8–49,1)	54,0 (43,3–64,4)
**Adequação segunda rotina** c		18,9 (15,8–22,5)	15,2 (12,6–18,2)	34,6 (25,8–44,6)
**Vacinação tétano**				
	Imunização adequada	59,9 (52,1–67,2)	61,5 (52,5–69,8)	52,9 (46,6–59,1)
	Imunização inadequada	5,0 (2,7–9,2)	5,4 (2,5–11,0)	3,7 (2,0–6,7)
	Sem informação	35,1 (29,7–40,9)	33,2 (27,3–39,6)	43,4 (36,4–50,6)
**Vacinação hepatite B**				
	Imunização adequada	35,8 (28,8–43,4)	36,1 (27,9–45,2)	34,5 (26,6–43,3)
	Imunização inadequada	15,2 (11,8–19,4)	16,6 (12,0–22,4)	9,5 (6,2–14,4)
	Sem informação	49,0 (42,8–55,2)	47,3 (40,3–54,5)	56,0 (47,4–64,3)
**Adequação vacinação**		31,6 (23,6–40,9)	31,8 (22,4–43,0)	30,6 (23,4–39,0)
	Suplementação com sulfato ferroso	39,8 (31,0–49,3)	43,2 (31,5–55,8)	25,2 (14,9–39,3)
	Suplementação com ácido fólico	33,7 (26,7–41,5)	36,4 (27,5–46,4)	22,3 (13,0–35,6)
**Adequação suplementação**		29,4 (22,0–38,1)	31,7 (22,3–42,9)	19,9 (11,2–32,8)
**Orientações**				
	Riscos e benefícios do parto vaginal	50,6 (44,3–56,9)	45,1 (39,2–51,2)	74,0 (58,1–85,4)
	Riscos e benefícios da cesariana	48,6 (42,2–55,0)	42,3 (36,7–48,0)	75,7 (62,5–85,3)
	Maternidade de referência	66,2 (55,1–75,7)	63,4 (50,3–74,8)	72,3 (53,4–85,6)
	Triagem tabagismo	68,7 (60,6–75,8)	67,4 (58,0–75,5)	74,3 (62,1–83,6)
	Triagem uso de álcool	72,6 (64,8–79,3)	71,0 (61,9–78,7)	79,6 (68,0–87,7)
**Adequação orientação**		17,6 (10,8–27,2)	11,9 (7,2–18,8)	41,8 (23,4–62,9)
**Adequação total** d		0,6 (0,3–1,5)	0,5 (0,2–1,5)	1,1 (0,3–4,2)

IC95%: Intervalo de confiança 95%. USG: ultrassonografia.

a Dados relativos apenas a mulheres que tiveram cartão de pré-natal digitado.

b Financiamento público: mulheres com parto em hospital público ou privado com internação paga pelo Sistema Único de Saúde; financiamento privado: mulheres com parto em hospital privado com pagamento por planos de saúde ou desembolso direto.

c Em mulheres com idade gestacional ≥ 34 semanas (público n = 1.047; privado n = 251; total n = 1.298).

d Mulheres com início do pré-natal até 12 semanas gestacionais; número adequado de consultas para a idade gestacional no parto; todos os exames de rotina (segunda rotina apenas para mulheres com idade gestacional ≥ 34 semanas); vacinação contra tétano e hepatite B; suplementações com sulfato ferroso e ácido fólico; e orientações sobre fumo, álcool, tipo de parto e maternidade de referência para internação para o parto.

A proporção de gestantes com ausência de informação sobre vacinação foi de 35,1% para o tétano e de 49,0% para hepatite B, tendo 59,9% dos cartões o registro de imunização adequada para o tétano e 35,8% para hepatite B, sem diferenças significativas segundo o tipo de financiamento (Tabela 3). A prescrição de sulfato ferroso estava presente em 39,8% dos cartões e a de ácido fólico em 33,7%, com valores mais baixos em mulheres com financiamento privado (Tabela 3). Metade das mulheres referiram ter recebido orientações sobre os riscos e benefícios do parto vaginal e da cesariana e 66,2% foram orientadas quanto à maternidade de referência para assistência ao parto. Aproximadamente 70% das mulheres referiram terem sido questionadas sobre tabagismo e consumo de álcool na gestação. Mulheres com financiamento privado receberam mais orientações sobre os riscos e benefícios dos tipos de parto, sem diferenças significativas em relação às outras orientações recebidas (Tabela 3).

A adequação do PN variou entre 79,3% (época de início) e 17,6% (orientações). Quando foram avaliados todos os componentes conjuntamente, a adequação atingiu apenas 0,6% das puérperas, sem diferenças significativas segundo o tipo de financiamento (Figura 2 e Tabela 3).

**Figura 2:** Adequação da assistência pré-natal segundo o tipo de financiamento do parto. Estado do Rio de Janeiro, 2021–2023.

## DISCUSSÃO

Os resultados deste estudo revelam baixa adequação do PN no ERJ, tendo apenas os indicadores "realização de pelo menos uma consulta de PN", "recebimento do cartão de PN" e "realização de uma USG na gestação" alcançado o valor superior a 95%, utilizado como padrão de referência. Ao se utilizar um conjunto de critérios, recomendados tanto pela OMS quanto pelo Ministério da Saúde brasileiro, a adequação foi observada em menos de 1% das gestantes. Os resultados demostram ainda a desigualdade social existente no ERJ, com maior proporção de gestantes adolescentes, de mulheres pardas e pretas, com menos anos de estudo, sem companheiro e sem trabalho remunerado entre aquelas com financiamento público, bem como desigualdades no acesso e utilização dos serviços de pré-natal dessas mulheres, com menor cobertura de assistência PN, início mais tardio, número inadequado de consultas e menor acesso a orientações e exames. Desigualdades sociais e demográficas na adequação do PN também foram verificadas em outros estudos brasileiros, com adequação mais baixa em mulheres pretas^12,18,19^, adolescentes^7,12,19^ e naquelas com menor escolaridade^7,12^ e renda^4,7^.

Não foram identificados estudos prévios que tenham avaliado a adequação do PN no ERJ. Ainda que não seja a comparação ideal, dados de estudo de âmbito nacional, realizado nos anos 2011/2012^20^ e que estimou resultados para a Região Sudeste, onde o ERJ está localizado, revelam aumento do número de puérperas que levaram o cartão da gestante para a maternidade — de 78,2% na Região Sudeste para 97,5% neste estudo. Foi constatado menor recebimento de cartões e de apresentação deles na maternidade por mulheres com financiamento privado, provavelmente pelo modelo de atenção predominante nesse setor, em que o acompanhamento PN e ao parto é geralmente realizado pelo mesmo profissional de saúde^21^.

Houve também aumento da proporção de gestantes com início da assistência pré-natal até a 12ª semana gestacional, ao compararmos com dados observados em 2011/2012, de 58,2% na Região Sudeste para 79,3% neste estudo^5^. Considerando-se o tipo de financiamento, a comparação de dados nacionais com dados deste estudo mostra aumento de 51,6 para 75,4% no público e de 60,3 para 95,7% no privado^5^. Dados do SINASC demonstram aumento do início precoce no ERJ no período 2012–2022, passando de 65,6 para 78,1%, sendo a média nacional de 80,8%^8^.

A comparação da adequação do número de consultas com outros estudos é limitada pelo critério utilizado neste estudo, de no mínimo oito consultas para uma gestante de risco habitual, com estudos anteriores que utilizavam geralmente o critério de seis consultas. Em 2011/2012, 73,1% das gestantes apresentaram o mínimo de seis consultas^20^, enquanto no terceiro ciclo de avaliação do Programa de Melhoria do Acesso e da Qualidade (PMAQ), realizado em 2017/2018, esse número foi de 77,8%^6^. Neste estudo, 63,3% das mulheres relataram oito ou mais consultas, uma adequação menor, porém com um critério mais exigente, ressaltando-se que 75,5% apresentaram número adequado de consultas para a IG no parto. Dados do SINASC mostram aumento do número de mulheres com mais de sete consultas no ERJ no período 2012–2022, passando de 54,9 para 64,3%, sendo a média nacional 63,7%^8^.

Com exceção da USG, nenhum dos exames de rotina alcançou a adequação desejada, com implicações negativas para o diagnóstico e tratamento de várias condições que afetam os desfechos maternos e perinatais, tais como infecção por sífilis e HIV, infecção urinária, diabetes e anemia. Adequação ainda menor foi observada para os exames da segunda rotina, mesmo considerando-se apenas as mulheres com 34 ou mais semanas gestacionais no parto. A maior adequação em mulheres com financiamento privado, também observada em estudo anterior^5^, possivelmente decorre de maior acesso a exames laboratoriais em usuários do setor privado. A adequação de exames, à exceção da USG, foi similar à observada em estudo nacional realizado em 2011/2012^20^ e inferior à observada no terceiro ciclo de avaliação do PMAQ^6^, ressaltando-se que neste último a fonte de informação foi entrevista com mulheres que tinham filhos com menos de dois anos, mais sujeita a viés de memória. Em relação a estudo com gestantes com assistência PN em unidades públicas no MRJ^13^, houve aumento da proporção de gestantes com financiamento público que realizaram o primeiro exame de urina e sorologia para HIV e das que realizaram o segundo exame de urina e sorologia para sífilis.

A vacinação durante a gestação é uma importante medida de prevenção do tétano neonatal e da transmissão vertical da hepatite B. A cobertura de vacinação antitetânica em mulheres com financiamento público foi inferior à observada no terceiro ciclo do PMAQ^6^ e em estudo com grávidas atendidas em unidades públicas do MRJ^13^, mas similar à relatada em estudo no Espírito Santo^4^, ressaltando-se proporção elevada de cartões sem registro de informação. Não foram identificadas pesquisas que tenham avaliado a vacinação para hepatite B em gestantes, com adequação de apenas um terço das puérperas avaliadas neste estudo. Em investigação realizada em João Pessoa/Paraíba, foi relatada vacinação adequada para tétano, hepatite B e influenza em 71% das gestantes^7^.

A suplementação com ferro e ácido fólico durante a gestação apresenta efeitos benéficos em populações de baixa e média renda, com redução da proporção de nascidos com baixo peso ao nascer e provável redução da proporção de nascimentos prematuros e de bebês pequenos para a idade gestacional^22^. A adequação da suplementação com SF foi muito inferior à observada no PMAQ^6^ e em estudo em unidades públicas no MRJ^13^, ressaltando-se que nesses estudos essa informação foi obtida em entrevista com a mulher, enquanto neste trabalho foi extraída do cartão da gestante. Não foram identificados trabalhos nacionais que tenham avaliado a suplementação com ácido fólico.

O PN é um momento privilegiado para a realização de ações educativas. Neste estudo, selecionamos quatro orientações pela sua relevância: triagem do tabagismo e uso de álcool na gestação, que são fatores de risco para desfechos perinatais negativos^23,24^ e modificáveis por meio de ações educativas e serviços de apoio^25^; informações sobre vantagens e desvantagens dos tipos de parto, visando à promoção do parto vaginal, num país que apresenta uma das maiores taxas de cesariana global; e vinculação da gestante à maternidade de referência para assistência ao parto, prevista por lei nacional desde 2007^26^. Ações de aconselhamento dependem apenas da atuação do profissional de saúde e podem ser realizadas em consultas individuais ou em grupo, tendo alcançado 2/3 das mulheres para triagem para fumo e álcool e orientações sobre maternidade de referência e apenas metade das mulheres para orientações sobre parto. Pesquisas anteriores que avaliaram a vinculação para maternidade de referência^13,20^ e algum tipo de orientação sobre parto (sinais do trabalho de parto, atividades para facilitar o parto)^7,13,20^ encontraram valores similares ao deste estudo. Não foram identificados trabalhos que tenham avaliado triagem para tabagismo e uso de álcool na gestação no Brasil, sendo a proporção de profissionais que referem realizar essas triagens rotineiramente variável entre países^27–29^.

A avaliação da adequação global da assistência PN foi inferior a 1%, com redução expressiva principalmente após a inclusão dos exames e das orientações. Resultado semelhante foi observado em estudos que incorporaram outros elementos além da época de início e do número de consultas^4,5,7,13,30^, com variação de 0,4^4^ a 38,5%^13^, sendo a comparação limitada pelos critérios utilizados. Cabe ressaltar a ausência de diferenças significativas em mulheres com financiamento público ou privado, mostrando os desafios para a prestação de assistência integral e abrangente em ambos os setores.

Este estudo apresenta algumas limitações. Foram analisadas apenas as mulheres que apresentavam cartão digitado, com perda de 20% das que o exibiram na maternidade. Essa perda foi seletiva, sendo maior em mulheres com financiamento privado, que tiveram maior adequação de alguns componentes analisados. Dessa forma, a adequação de PN do total de mulheres do ERJ pode estar subestimada. Mulheres com parto domiciliar, em via pública ou em hospitais com menos de cem nascimentos/ano não foram incluídas neste estudo, e os resultados apresentados não refletem a assistência PN desse grupo de mulheres. Dados sobre exames, vacinas e suplementações foram obtidos no cartão da gestante e esses procedimentos e prescrições podem ter sido realizados e não registrados. Entretanto, o cartão da gestante é o principal elo entre a assistência ambulatorial e hospitalar, sendo o registro de informações também uma medida de adequação do PN. Por fim, o financiamento da assistência ao parto foi utilizado para a classificação das mulheres nos setores público e privado, sendo possível que o tipo de financiamento tenha sido diferente para a assistência PN e ao parto, principalmente para aquelas que apresentam cobertura do plano de saúde apenas para atendimento ambulatorial, o que pode ter atenuado a diferença entre os grupos.

A assistência PN no ERJ mostrou-se inadequada quando foram considerados todos os itens analisados. A menor adequação de vários componentes do PN em mulheres com financiamento público, que constituem um grupo com maior vulnerabilidade social, aumenta a probabilidade de desfechos negativos nessa população. Torna-se essencial a implantação de estratégias destinadas a oferecer o melhor cuidado justamente àquelas que mais necessitam dessas ações.

## Data Availability

Os conjuntos de dados gerados e/ou analisados durante o presente estudo não estão publicamente disponíveis devido a restrições [éticas/legais/de privacidade], mas estão disponíveis com o autor correspondente mediante solicitação.
